# Exploring the Optimal Timing of Endoscopic Ultrasound Performance Post-Acute Idiopathic Pancreatitis

**DOI:** 10.3390/diagnostics12081808

**Published:** 2022-07-27

**Authors:** Tawfik Khoury, Amir Shahin, Wisam Sbeit

**Affiliations:** 1 Galilee Medical Center, Gastroenterology, Nahariya 2220903, Israel; amirs3@gmc.gov.il (A.S.); wisams@gmc.gov.il (W.S.); 2 Azrieli Faculty of Medicine, Bar-Ilan University, Safed 5290002, Israel

**Keywords:** EUS, timing, idiopathic, pancreatitis, severity, scores

## Abstract

Background: Patients with acute idiopathic pancreatitis (AIP) should undergo further imaging tests such as endoscopic ultrasound (EUS) for further investigation. The time interval between an episode of AIP and EUS performance is still controversial. Aims: We aimed to explore the optimal timing for performing EUS and to reveal parameters that might predict longer intervals needed for performing EUS. Methods: We performed a single-center retrospective study at Galilee Medical Center from January 2015 to January 2020, at which point we included all patients who underwent EUS for further investigation of AIP. Results: Overall, we included 50 patients. The average age of all patients was 54.2 ± 17.6 years (range 22–69 years), and more than half of the study cohort were males (58%). Classifying patients as inflamed vs. normal pancreatic tissue on EUS, we found that among patients with normal pancreatic tissue, EUS was performed 44.7 ± 28.3 days from discharge, while for patients with inflamed pancreatic tissue, it was 48.1 ± 22.3 days (*p* = 0.37) after discharge. Notably, the CT severity index was significantly associated with inflamed pancreatic tissue on EUS, as it was 2.4 ± 0.74 vs. 1.5 ± 1.3 in the normal pancreatic tissue group (*p* = 0.03). There were no differences in the Bedside index for severity in acute pancreatitis (BISAP) scores, and there were no differences in the average American Society of Anesthesiologist Physical Status (ASA) scores between the two groups. Notably, 26.3% of patients had inflamed pancreatic tissue when performing EUS at 4 weeks, as compared to 16% who had inflamed pancreatic tissue at EUS performed after 6 weeks. Conclusion: Radiological severity score was the only important factor in determining the time interval of performing EUS after an episode of AIP. Intervals greater than six weeks seem to be needed among patients with higher Balthazar scores.

## 1. Introduction

Acute pancreatitis is defined as a sudden inflammation of the pancreas. It is characterized by the presence of two out of three parameters (typical abdominal pain, elevated serum amylase or lipase at least three times the upper normal limit, and abdominal imaging consistent with acute pancreatitis) and can be a consequence of numerous causes [1]. Among them, gallstone disease and alcohol are the most common causes in about 42% and 21% of patients, respectively [2].

Most causes of acute pancreatitis can be diagnosed based on clinical history, physical examination, routine laboratory studies, and conventional radiologic methods (transabdominal ultra-sonography (US) and computed tomography (CT)). However, in certain cases where no underlying cause is identified by standard investigation, the patients are classified as having acute idiopathic pancreatitis (AIP) [3,4].

AIP is responsible for almost 10–30% of cases of acute pancreatitis [5,6]. Patients with AIP are defined as patients with a confirmed diagnosis of acute pancreatitis with normal standard investigations, including the absence of clinical causes (alcohol consumption, smoking, and medications), laboratory studies (serum triglyceride and calcium level), and the absence of biliary and pancreatic pathology using US and CT [6].

The professional guidelines recommend repeating abdominal imaging after discharge [7,8], as the repeated imaging study increases the diagnostic yield by approximately 20% for the identification of microlithiasis and sludge [9], which are generally responsible for the major causes of presumed AIP [10,11]. Therefore, when previous diagnostic investigations fail to reveal an etiology, further advanced imaging studies are needed to accurately diagnose the cause of AIP; this allows for proper treatment and may prevent the high recurrence rate of AIP, and its associated morbidity and mortality. Notably, the recurrence rate may reach 30%, while the diagnosis remains obscure in almost 30% of cases [5]. To further investigate the etiology of AIP, several modalities have been used, including endoscopic ultrasound (EUS) and magnetic resonance cholangio-pancreatography (MRCP). Several guidelines have recommended EUS as the first diagnostic step in presumed AIP for the diagnosis of other diseases causing pancreatitis such as biliary disease, neoplasms, and chronic pancreatitis [7,8,12,13], followed by secretin-enhanced MRCP to identify rare morphological abnormalities [7,14]. EUS has been studied as a modality for elucidating the etiology of acute idiopathic pancreatitis, which showed a yield in about 50–80% of patients [15,16,17]. However, the timing of performing EUS after an episode of acute idiopathic pancreatitis is still unclear given the fact that the pancreatic inflammation may lead to missed lesions and impact the safety of the procedure [18]. Different studies have used different timings [19]. In a recent prospective study of patients with AIP, EUS was performed 1 month or more after hospital discharge [20]. To the best of our knowledge, to date, no studies have assessed pancreatic tissue appearance at EUS at a specific time interval after hospital discharge from an episode of acute pancreatitis. Therefore, we aimed to assess the optimal timing of EUS performance after an episode of AIP.

## 2. Study Design

This is a retrospective single-center study with inclusion of all patients who were hospitalized with AIP and who underwent EUS as a part for their standard investigations at Galilee Medical Centre, Nahariya, from January 2015 to January 2020. The inclusion criteria included patients older than 18 years of age who were hospitalized with AIP and who had undergone EUS as part of their investigations (following an unrevealing abdominal ultrasound). The exclusion criteria included patients with known hepato-biliary diseases such as chronic pancreatitis, autoimmune pancreatitis, pancreatic cystic lesions, pancreatic cancer, pancreatic neuro-endocrine tumors, pancreatic duct stones, gallbladder and common bile duct stones, autoimmune cholangitis, and biliary malignancy and patients with active heavy alcohol consumption defined as 14 drinks or more for men, and 7 drinks or more for women per week; patients who drank in moderation in the last year before the end date of patient enrollment (from January 2019 to January 2020) were included, with moderation defined according to the “Dietary Guidelines for Americans 2020–2025” as an intake of two drinks or less in a day for men, and one drink or less in a day for women [21]. After a thorough review of the medical documentation and charts of patients, demographic data (age and gender), clinical parameters (body mass index, obesity, alcohol consumption and smoking habits defined as any active smoking {measured by pack years}), clinical (BISAP and ASA) and radiological (CT severity index) scores of the severity of acute pancreatitis were extracted. Moreover, we assessed the timing of EUS performance after an episode of acute pancreatitis and assessed endosonographic pancreatic appearance as either normal or inflamed. All procedures were carried out via linear echoendoscope (Pentax-Japan), model 3870, and performed by a single endosonographser (WS) with more than 15 years of experience in the field of endoscopic ultrasound. Patients were placed in the left lateral decubitus position and were sedated with intravenous midazolam and propofol according to the decision of the endoscopist. The study protocol conformed to the ethical guidelines of the 1975 Declaration of Helsinki and was approved by the local institutional ethics committee. Written informed consent was waived due to the retrospective study design.

## 3. Clinical and Radiological Scores That Were Assessed in Our Cohort

Clinical severity was assessed by three severity scores. *The Atlanta severity score* defined as (0) mild acute (absence of organ failure), (1) moderate (transient organ failure that resolved within 48 h or local or systemic complications not more than 48 h), or (2) severe acute (persistent organ failure) [22]. *The* *Bedside index for severity in acute pancreatitis (BISAP) score* including 5 parameters: blood urea nitrogen (BUN > 25 mg/dl—1 point), impaired mental status (Glasgow coma score < 15—1 point), systemic inflammatory response syndrome (SIRS—1 point), age (>60 years—1 point), and pleural effusion (1 point). A BISAP score of 0–2 points indicates lower mortality of <2%, and a score of 3–5 indicates higher mortality of >15% [23]. *The* *American Society of Anesthesiology (ASA) score* was used to determine the patient tolerability to surgery and anesthesia by assessing comorbidities, which include 6 grades: (1) normal health, (2) mild systemic disease, (3) severe systemic disease, (4) severe life-threatening systemic disease, (5) a moribund patient who is not expected to survive without the operation, or (6) a patient with brain death. Moreover, the CT severity index was assessed and defined by the 5 radiological grades: (A) normal pancreas (0 points), (B) enlargement of pancreas (1 point), (C) inflammatory changes in pancreas and peripancreatic fat (2 points), (D) ill-defined single peripancreatic fluid collection (3 points), and (E) two or more poorly defined peripancreatic fluid collections (4 points). A score of 0–3 indicates mild pancreatitis with a 3% mortality rate, a score of 4–6 points indicates moderate pancreatitis with a mortality rate of 6%, and a score of 7–10 indicates severe pancreatitis with 17% mortality rate [24].

## 4. Statistical Analysis

The characteristics of participants were presented as descriptive statistics. Continuous variables with normal distributions were presented with informative statistics, such as arhythmical means and standard deviations (±SD) according to the Kolmogorov–Smirnov test. Categorical variables were presented as numbers and percentage tables. Figures with *p*-values less than 0.05 were considered statistically significant. Statistical analyses were carried out with commercial software, Statistical Package for Social Science (SPSS version 24.0, IBM, Chicago, IL, USA).

## 5. Results

### 5.1. Demographics and Baseline Characteristics

**Figure 1:**
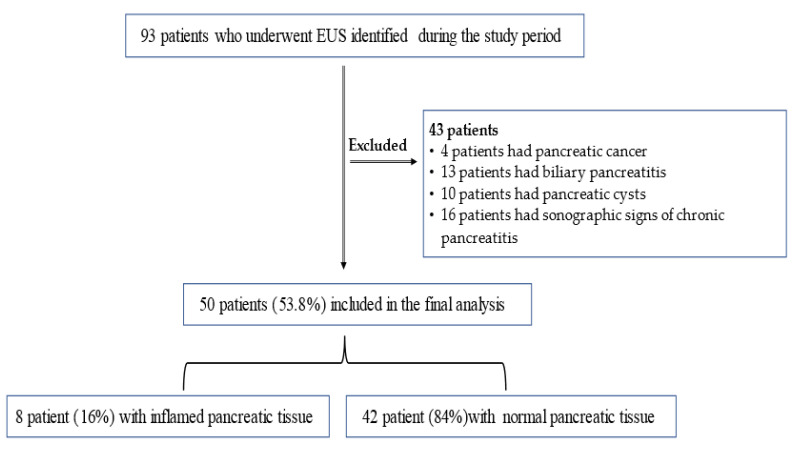
The study flowchart.

Overall, 93 patients were identified during the study period. Among them, 43 patients were excluded (4 had pancreatic masses, 13 had biliary pancreatitis, 10 had pancreatic cysts, and 16 had sonographic signs of chronic pancreatitis), while the remaining 50 patients with AIP were included in the final analysis. Figure 1 demonstrates the study flowchart. The average age of the entire cohort was 54.2 ± 17.6 years (range 22–69), and 29 patients (58%) were males. The average body mass index (BMI) was 29 ± 5.5 (19.7–43.4). Notably, mild, moderate, and severe pancreatitis were present in 41 patients (82%), 7 patients (13.7%), and 2 patients (3.9%), respectively. The average BISAP score was 0.78 ± 0.94 (0–4), and the average ASA score was 1.83 ± 0.56 (1–3). Additionally, the pancreatitis severity score per CT according to the CT severity index was 1.9 ± 1.1 (0–4). Table 1 demonstrates the demographics and clinical characteristics.

### 5.2. Comparison between Patients with Inflamed vs. Normal Tissue on EUS

**Figure 2:**
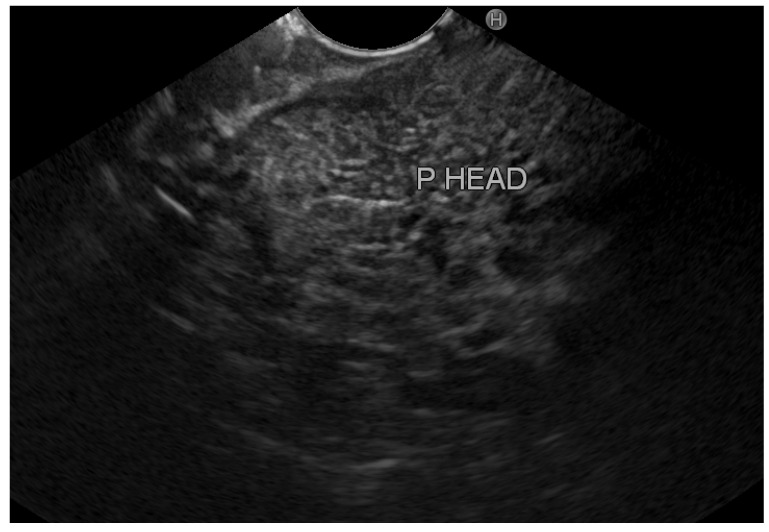
Inflamed pancreatic parenchyma. P HEAD: pancreatic head.

**Figure 3:**
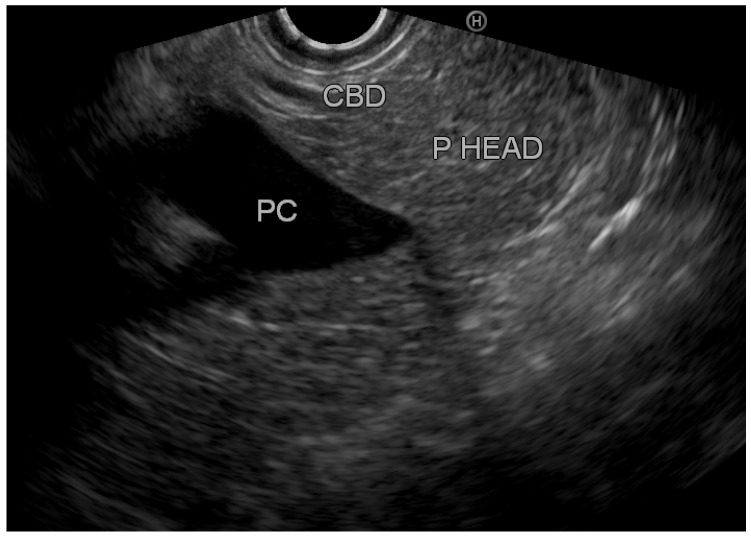
Normal pancreatic parenchyma. CBD: common bile duct; PC: portal confluence; P HEAD: pancreatic head.

Overall, we identified eight patients with inflamed tissue on EUS (group A) vs. 42 patients with normal pancreatic tissue (group B). Figure 2 demonstrates an EUS image with inflamed pancreatic head tissue (heterogeneous echo pattern, decreased echogenicity with edema), and Figure 3 demonstrates normal pancreatic head parenchyma. All patients in group A had clinically mild acute pancreatitis. However, in group B, 33 patients (78.6%) had mild disease, 7 patients (16.7%) had moderate disease, and 2 patients (4.8%) had severe disease. Exploring the optimal timing for performing EUS, we found that among patients with normal pancreatic tissue, the average period until performing EUS from hospital discharge was 44.7 ± 28.3 days (20–120); among patients with inflamed pancreatic tissue, EUS was performed 48.1 ± 22.3 days (20–90) from discharge. Table 2 demonstrates the characteristics of patients with inflamed and normal pancreatic tissue on EUS.

### 5.3. Exploring the Optimal Timing according to Clinical and Radiological Severity Scores

The BISAP score was significantly lower in group A, as it was 0, compared to 0.95 ± 0.96 in group B (*p* = 0.03). There was no difference in the average ASA score, as it was 1.6 ± 0.52 in group A vs. 1.9 ± 0.57 in group B (*p* = 0.13). Interestingly, the CT severity index was significantly higher in group A, 2.4 ± 0.74, than it was in group B, 1.5 ± 1.3 (*p* = 0.03), suggesting that the CT severity index is more important than the other clinical scores in determining the timing of EUS performance.

**Figure 4:**
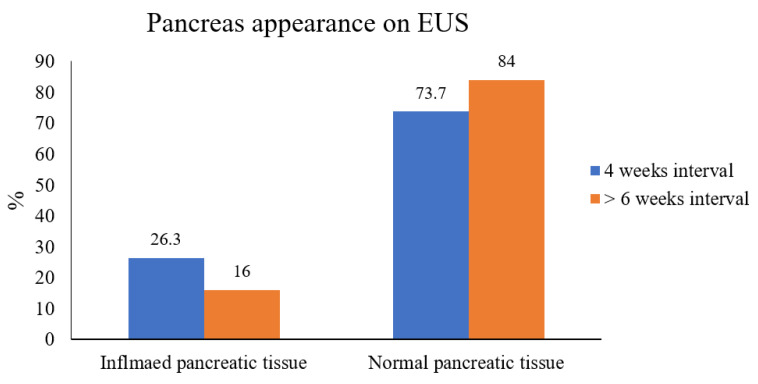
EUS pancreatic tissue appearance according to time interval.

In calculating the time for performing EUS after discharge from hospitalization due to acute idiopathic pancreatitis hospitalization, we found 19 patients who underwent EUS up to 4 weeks after discharge; among them, 5 patients (26.3%) still had inflamed pancreatic tissue on EUS, as compared to 14 patients (73.5%) who had normal pancreatic parenchyma. Notably, only the CT severity index was higher in the inflamed pancreatic tissue group, with an average of 1.8 ± 1.1, compared to 1.4 ± 1.5 among patients with normal pancreatic tissue, with a statistically insignificant *p* value of 0.3. Further analysis on a time interval of more than 6 weeks before performing EUS after an episode for acute pancreatitis, we found that only 8 patients (16%) still had inflamed pancreatic tissue on EUS, while 42 patients (84%) had a normal appearing pancreas corresponding to a CT severity index of 2.4 ± 0.074 vs. 1.5 ± 1.3, respectively, with a significant *p* value of 0.03 (Figure 4). This suggests a cut-off period of more than 6 weeks before performing EUS after a discharge from an episode of acute idiopathic pancreatitis.

## 6. Discussion

Not infrequently, the underlying cause of an acute pancreatitis episode remains obscure even after standard clinical, biochemical, and radiological (US and CT) evaluation, creating a subgroup of acute pancreatitis referred to as acute idiopathic pancreatitis (AIP) [25]. Previous studies have shown that an EUS examination might be beneficial in exploring the underlying cause of a presumed AIP [26]. During an episode of AIP, the yield of EUS in exploring the pancreatic parenchyma and ducts is very limited given the presence of edema of the duodenal wall, pancreatic inflammation, necrosis, or peri-pancreatic fluid collection, as these can deeply hinder the visualization of the pancreas, and occasionally the gallbladder or bile duct. This hindered visualization can significantly affect the diagnostic accuracy of EUS [27]. The time interval for the performance of EUS after AIP is controversial given concerns that inflammation from acute pancreatitis may lead to missed lesions and impact the safety of the procedure [18,19]. Two studies have suggested performing an EUS examination at least four weeks after an episode of acute pancreatitis [20,28]. In other studies, endosonographers frequently performed EUS examinations between four and eight weeks after the clinical presentation of AIP, based on expert opinion [19,29]. The rationale behind performing EUS at least 4 weeks after the initial presentation of acute pancreatitis is the fact that there are inflammation and/or necrosis within the pancreatic parenchyma that prevent accurate visualization and assessment of pancreatic lesions during the acute phase [29]. However, the main drawback of postponing EUS is the missed or delayed diagnosis of occult pancreatic and biliary malignancies [18]. Due to this detriment of occult malignancies, the timing of EUS should be clearly defined, to perform a good quality EUS exam from one side, and not to postpone EUS for long time and miss pancreato-biliary tumors on the other side. Currently, there is a controversy in the literature regarding the timing of performing EUS after an episode of acute idiopathic pancreatitis [30,31]. In our study, the mean time from discharge until EUS performance was 47.3 ± 30.7 days in the entire cohort. For patients with normal pancreatic parenchyma on EUS, the mean time from discharge to the performance of EUS was 44.7 days, suggesting that more than 6 weeks is an adequate time before performing EUS. Among patients with inflamed pancreatic tissue, the average time interval was 48.1 days. Additionally, we found that the clinical pancreatitis severity scores did not affect the timing of performing EUS. However, the CT severity index was higher in patients with inflamed pancreatic tissue according to EUS, suggesting that among patients with more severe pancreatitis on imaging (higher CT severity index), a longer interval of at least 6 weeks post AIP might be needed before performing an EUS examination. Patients with a lower CT severity index of no more than 2.4 according to our results (as the CT severity index is defined by integers, the lower score could be translated into scores 0 and 1, but an accurate CT severity index threshold needs to be defined by other studies) may benefit from earlier EUS study. It could uncover the etiology of AIP and prevent delay in diagnosis, as there is the potential of recurrent acute pancreatitis with potential complications, or a delay in discovering malignancy beneath the episode of AIP. A thorough search of the literature did not yield publications exploring the correlation between EUS timing post AIP and the CT severity index; however, several studies have reported the timeline interval for the performance of EUS after an episode of AIP [19]. A previous prospective study by Wilcox et al. reported that EUS was performed 4 weeks or more after hospital discharge; however, this study did not address whether the time interval used was sufficient for adequate pancreatic tissue evaluation [20]. Moreover, Rana et al. and Yusoff et al. reported the performance of EUS at least 4 weeks after an episode of AIP, again without addressing the appearance of pancreatic parenchyma on endosonography [28,31]. On the other hand, Thevenot et al. reported the yield of EUS vs. MRCP performed 8 weeks after AIP, however, without commenting on the pancreatic appearance on sonography [29].

The limitations of our study are its retrospective and single-study design. The other limitations are the small number of patients included with inflamed pancreatic tissue on an EUS examination and the fact that we did not have radiological and clinical follow-up after the patients with inflamed pancreatic tissue to assess whether their inflammation resolved or continued. On the other hand, the strength of our study is that the pancreatic tissue appearance was assessed by EUS, which is the optimal imaging modality for examining the echogenicity of the pancreatic parenchyma, making our results reliable.

In conclusion, the timing of performing EUS after an episode of acute pancreatitis was mainly based on the radiological pancreatitis severity according to the CT severity index but not on the clinical pancreatitis severity score, as an interval larger than 6 weeks is needed before performing EUS among patients with a high CT severity index. Further prospective studies are needed to confirm our findings.

## Figures and Tables

**Table 1:** Demographics and clinical characteristics of the entire cohort.

Number of Patients	50
Age, mean ± SD (years)	54.2 ± 17.6
Male gender, N (%)	29 (58)
BMI, mean ± SD	29 ± 5.5
Obesity, N (%)	18 (36)
Smoking, N (%)	21 (42%)
Packs per year smoking, mean ± SD	38.3 ±15.1
Alcohol drinking in moderation, N (%)	2 (4)
Atlanta clinical severity of pancreatitis, N (%)	
Mild Moderate Severe	41 (82) 7 (14) 2 (4)
BISAP score, mean ± SD	0.78 ± 0.94
ASA score, mean ± SD	1.83 ± 0.56
CT severity index, mean ± SD	1.6 ± 1.3
Inflamed pancreatic tissue, N (%)	14 (28)
Time from discharge until EUS performance (days)	47.3 ± 30.7

**Table 2:** Characteristics of patients with inflamed and normal pancreatic tissue on EUS.

	Inflamed Pancreatic Tissue (Group A)	Normal Pancreatic Tissue (Group B)	*p* Value
Number of patients	8	42	-
Male gender, N (%)	6 (75)	22 (52.4)	0.12
BMI, mean ± SD	31.4 ± 6	28.7 ± 5.4	0.11
Obesity, N (%)	5 (62.5)	13 (30.9)	0.04
Smoking, N (%)	5 (62.5)	15 (36.6)	0.12
Packs per year smoking, mean ± SD	24.2 ± 14.5	40.8 ± 19.3	0.01
Alcohol drinking in moderation, N (%)	1 (12.5)	1 (2.4)	0.09
Atlanta clinical severity of pancreatitis, N (%)			
Mild Moderate Severe	8 (100) 0 0	33 (78.6) 7 (16.7) 2 (4.8)	-
BISAP score, mean ± SD	0	0.95 ± 0.96	0.003
ASA score, mean ± SD	1.63 ± 0.52	1.87 ± 0.57	0.13
CT severity index, mean ± SD	2.4 ± 0.74	1.5 ± 1.3	0.03
Time from discharge until EUS performance (days)	48.1 ± 22.3	44.7 ± 28.3	0.37

## Data Availability

The data are available at the gastroenterology department, Galilee Medical Center, and will be available upon a reasonable request.

## Abbreviations

AIP	acute idiopathic pancreatitis
EUS	endoscopic ultrasound
BISAP	Bedside index for severity in scute pancreatitis
ASA	American Society of Anesthesiologist Physical Status
US	ultrasound
CT	computed tomography
MRCP	magnetic resonance cholangio-pancreatography
BMI	body mass index
